# Massive Hemoptysis: An Unusual Complication of Left Atrial Appendage Occlusion Device in Two Patients

**DOI:** 10.7759/cureus.61451

**Published:** 2024-05-31

**Authors:** Yogita Mosalpuria, Muhammad Ibrahim, Mohamed Fayed, Jayakar Guruswamy, Alexandra R DePorre

**Affiliations:** 1 Anesthesiology, Pain Management and Perioperative Medicine, Mount Sinai Hospital, New York, USA; 2 Anesthesiology, Henry Ford Health System, Detroit, USA; 3 Anesthesiology, Montefiore Medical Center, Wakefield Campus, New York, USA

**Keywords:** ct angio, non valvular atrial fibrillation, massive hemoptysis, pulmonary venous hemorrhage, left atrial appendage occlusion

## Abstract

Left atrial appendage occlusion (LAAO) devices have emerged as a promising alternative for stroke prevention in non-valvular atrial fibrillation (NVAF) patients with contraindications to chronic anticoagulation therapy. The most common life-threatening procedural complications described in the literature include pericardial effusion, air embolism, and stroke. We here present a case report of two patients who experienced identical but rare post-procedural complications of pulmonary venous bleed, presenting as hemoptysis.

## Introduction

Atrial fibrillation is the most common sustained cardiac arrhythmia, occurring in 1-2% of the general population [1]. Clinically, it is associated with an increased incidence of death, stroke, left ventricular dysfunction, heart failure, thromboembolic events, hospitalization, and reduced quality of life [2]. Left atrial appendage occlusion (LAAO) devices have emerged as an appealing stroke prevention strategy in patients with non-valvular atrial fibrillation (NVAF) and who are poor candidates for lifelong anticoagulation therapy [3-4]. Complications that occur during LAAO device insertion procedures have been attributed to the close anatomical proximity of the left atrial appendage (LAA) to adjacent vessels and valves in the heart. In this complex spatial orientation, the pulmonary artery is anterosuperior to the LAA, the left superior pulmonary vein is posterior to the LAA, the mitral valve is inferior to the LAA, and the LAA itself covers an area above the atrioventricular groove, which is where the left circumflex artery and the great cardiac vein are located [5]. Here, we present a case report describing  two  patients who experienced hemoptysis due to pulmonary venous hemorrhage within one hour of an LAAO device insertion procedure. Our report describes two unique scenarios of this rare complication and successful management approaches, which will be critical for surgeons to be familiar with, as LAAO devices become more commonly used.

## Case presentation

An 88-year-old man who had a medical history of atrial fibrillation (CHA2DS2-VASc score 5, previously on anticoagulation therapy), hypertension, stroke, and tobacco smoking (57 pack-years) presented for an LAAO device insertion because of his history of persistent hematuria that required prostate fulguration, blood transfusions, and cessation of anticoagulation therapy. An 88-year-old woman with a medical history of atrial fibrillation (CHA2DS2-VASc score 4, previously on anticoagulation therapy), chronic obstructive pulmonary disease, myocardial infarction, ischemic cardiomyopathy (ejection fraction 51%), a previous drug-eluting stent in the right coronary artery, tachy-brady syndrome with a permanent pacemaker in situ, and mitral regurgitation presented for an LAAO device insertion because of her history of intermittent gastrointestinal bleeding requiring repeated hospitalizations for blood transfusion.

Both patients underwent general anesthesia with endotracheal intubation, induction of anesthesia with propofol/fentanyl/rocuronium, and maintenance with isoflurane and air/oxygen combination.  Both patients had an uneventful intraoperative course. Venous access was obtained via the femoral vein using ultrasonography and fluoroscopy. The transseptal puncture was performed using an SL1 catheter (transseptal guiding introducer) under transesophageal echocardiogram (TEE) and fluoroscopic guidance. Through the SL1 catheter, a multipurpose angiographic catheter was used to direct a J-wire into the left upper pulmonary vein, which was then exchanged for an Amplatz Super Stiff guidewire.  With an anterior curve delivery sheath positioned into the distal appendage over a pigtail catheter, an LAAO device (WATCHMAN FLX) was successfully deployed in the LAA for both patients. Prior to patient discharge, the Patient Acceptable Symptom State (PASS) criteria were checked for both patients (Position: device at LAA ostium; Anchor: fixation anchors engaged/device is stable; Size: device is compressed 8-20% of the original size; and, Seal: device spans ostium, all lobes of LAA are covered). No significant peri-device leakage was observed as seen by TEE and angiogram.  The flow across the transseptal defect was left to right and was not closed. 

**Figure 1:**
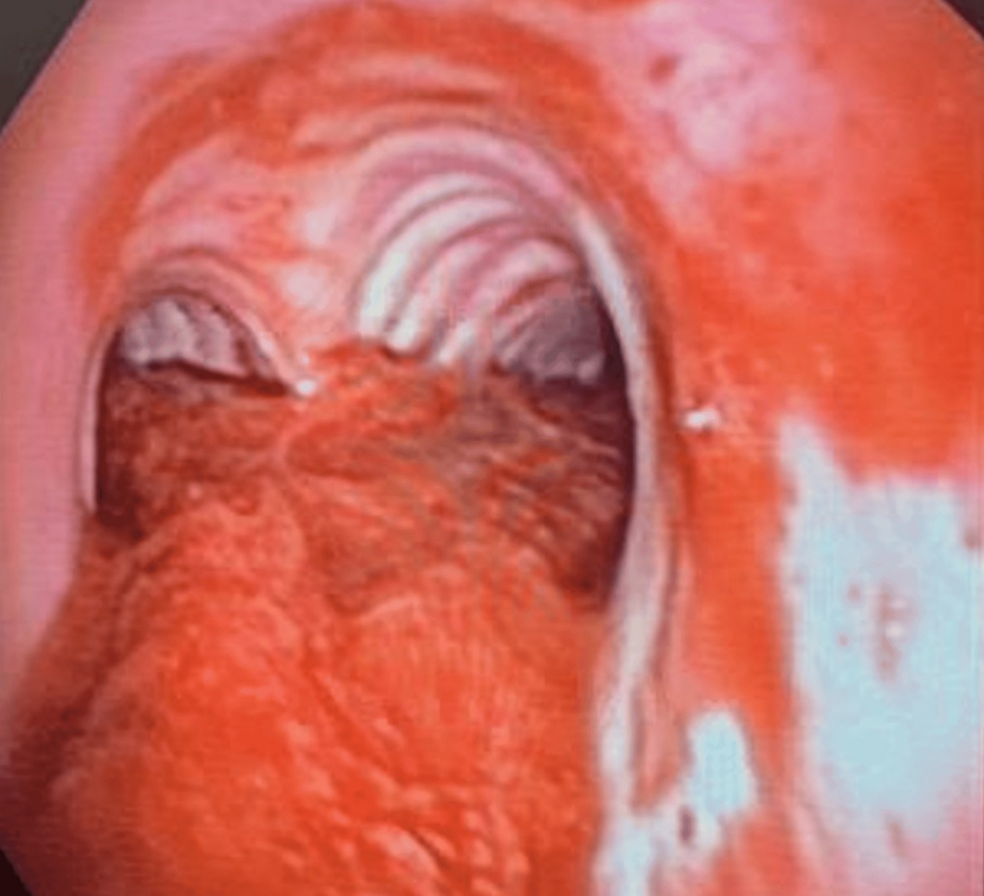
Bronchoscopy revealed saddle clot straddling carina (Patient One). No active bleeding noted. Due to coughing, the procedure was stopped.

**Figure 2:**
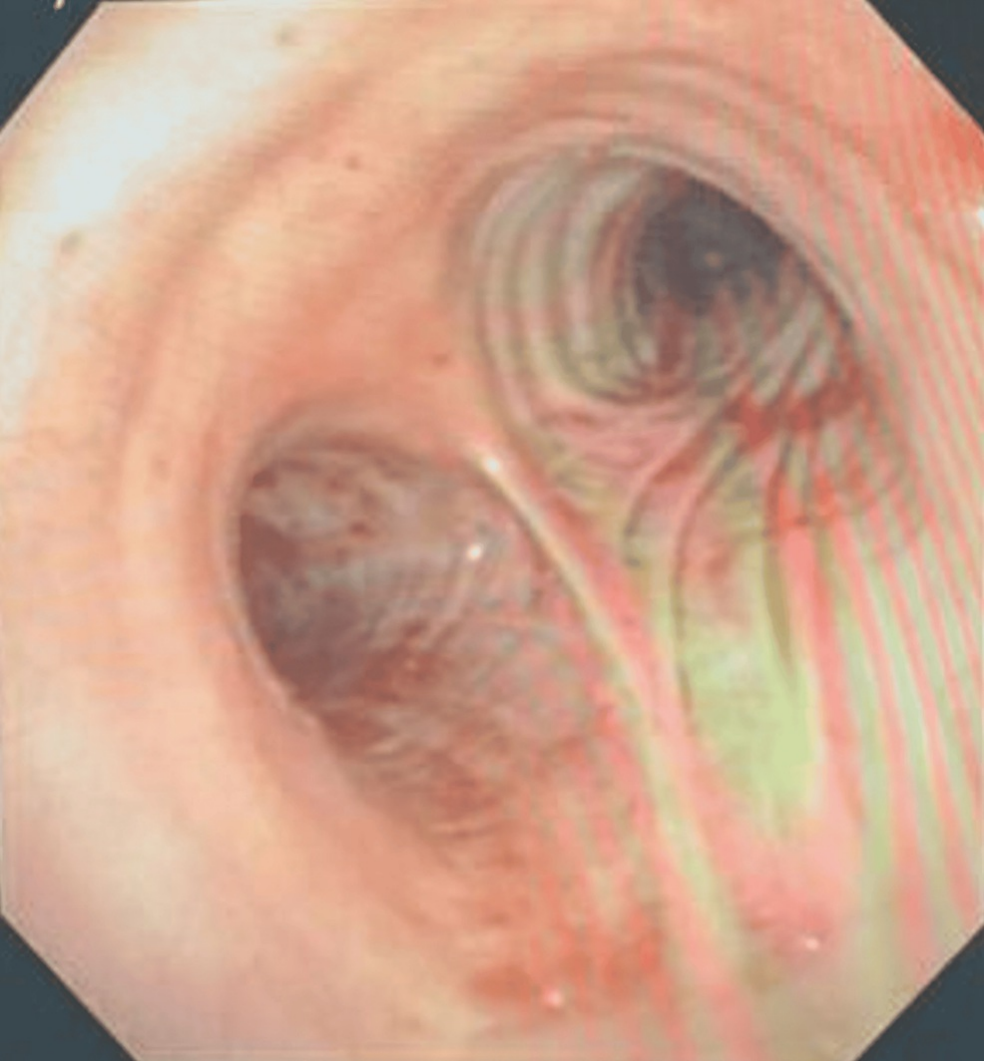
No active bleeding (Patient One). No endobronchial lesions.

Just prior to extubation and during emergence, patient one developed massive hemoptysis via the endotracheal tube. Despite repeated endotracheal suctioning, bleeding continued. In lieu of ongoing bleeding, a decision was made to keep the patient intubated, anesthesia maintenance switched to propofol infusion, and maintenance of hemodynamics with vasopressors and fluids as needed. Interventional pulmonology and thoracic surgery were consulted, and both teams arrived urgently at the catheterization lab. First, flexible bronchoscopy was performed by the pulmonology team which revealed a thrombus in the airways that was more extensive on the left side and extended over the carina with no active bleeding (Figure 1). Respiratory failure progressed as the patient required high oxygen and was unable to tolerate apneic periods. Patient One was admitted to the intensive care unit for elective ventilation, where he remained hemodynamically stable during his stay. On laboratory evaluation, his hemoglobin had dropped from 14 g/dL to 12.3 g/dL.  The patient thereafter underwent repeat bronchoscopy by the interventional pulmonology team. Blood clots were suctioned out (minimal use of forceps) throughout the entire left-sided airway and most of the right-sided airways, with the exception of some portion of the right middle and right upper lobes, with no active bleeding. Cryoablation was not required. Toward the end of the procedure, some oozing was noted from the lingula for which a scope was wedged and suction was held for 2 minutes. No further bleeding was noted (Figure 2). To identify the source of bleeding, a computed tomography (CT) angiogram of the chest was done, which showed dependent lung opacity (basilar; left greater than right lung), with no evidence of recurrent active bleeding within the trachea. The patient was extubated and discharged home two days later. 

**Figure 3:**
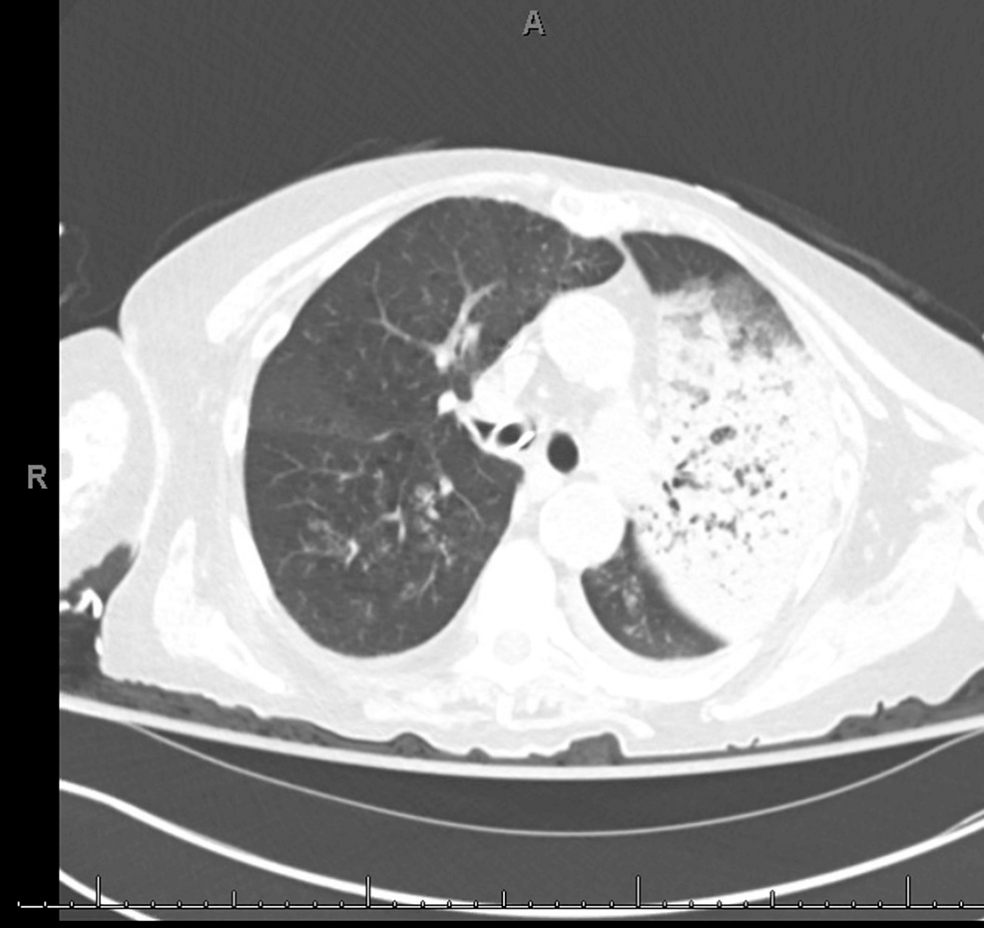
Non-contrast CT scan showing hemorrhage within left lung field (Patient Two).

Patient Two was extubated uneventfully at the end of the device insertion procedure and was transferred to the recovery area. Approximately one hour later, she had hemoptysis of bright red blood with associated hypoxia (spO_2_ 85-88% on a non-rebreather mask @10 L/min, 100% FiO_2_). Patient two continued to remain alert, conscious, and hemodynamically stable. In lieu of suspected residual heparinization, activated clotting time was rechecked and noted to be 200 sec, which was reversed with protamine. An otolaryngology team was consulted to rule out an upper pharyngeal or nasopharyngeal source of bleeding from possible unrecognized damage during TEE probe placement. Flexible laryngoscopy revealed no damage to the upper airway, although blood was seen coming up from the lower airway and collecting at the glottis. Given her persistent hemoptysis, patient two was intubated with a 9.0 mm endotracheal tube. Bronchoscopy was attempted through the endotracheal tube but was unsuccessful because of continued bleeding from the lung. The interventional pulmonology team was emergently consulted, and they performed a flexible bronchoscopy that showed significant bleeding originating from the left lingula. Multiple attempts of cold saline washing, continued suction, and wedging at the lingula did not stop the bleeding, and therefore, the endotracheal tube was advanced into the right mainstem bronchus for lung isolation. To determine the source of bleeding, patient two underwent an emergent non-contrast CT (NCCT) scan of the chest. Throughout the process, she continued to remain hemodynamically stable. NCCT showed filling defects within the left lingula and left lower lobe bronchial tree as well as near complete hyperdense consolidation of the left upper lobe (Figure 3). Contrast was not used during the CT scan to avoid the risk of contrast-induced nephropathy, since the patient had already received a significant amount of contrast during the device insertion procedure. Because of the patient’s continued venous bleeding, a decision was made to selectively ventilate the right lung overnight with a planned repeat bronchoscopy the following day. Bronchoscopy performed the next day showed cessation of bleeding with a thrombus occluding the lingula, which was suctioned and removed. The patient’s clinical condition remained stable, and she was extubated the following day with no long-term sequelae. A repeat TEE showed a well-positioned device in the LAA. Two months after the incident, a follow-up CT angiogram of the chest showed a minor LAA residual defect without pulmonary vascular stenosis or injury.

## Discussion

Massive hemoptysis is the term used to describe a large amount of expectorated blood or a rapid rate of bleeding from the lungs. While the term “massive” gives the impression that this complication, in and of itself, is associated with a serious risk of mortality [3]. The causes of hemoptysis are many, and in our patients, the likely cause was pulmonary vein irritation/micro-perforation from the Super Stiff guidewire used during LAAO device insertion [1].

Complications from LAAO device insertion procedures include damage to the contiguous valves and vascular structures, commonly causing pericardial tamponade [6]. Micro-perforation of the LAA from the hooks and struts of the LAAO device has been associated with delayed presentation of pericardial effusion and tamponade. In a multicenter study of the AMPLATZER cardiac plug in 1047 patients, pericardial tamponade was noted in 1.2% (13/1047), with one case reported as being caused by pulmonary artery tear (0.09%) [7-8]. We presume that the hemoptysis in our patients was due to pulmonary vein injury. Using a pre-shaped wire that remains in the left atrium may obviate the need for placement of a wire in the pulmonary vein, although there is no evidence that this is indeed safer than using a wire in the pulmonary vein.

The differential diagnosis for pulmonary hemorrhage also includes supraglottic airway injury from endotracheal tubes or TEE probes and gastrointestinal sources of bleeding. Rapid assessment of the patient to delineate the source of bleeding is critical. In intubated patients, bleeding within the endotracheal tube can easily be identified as originating from the bronchial tree. Examination of the oropharynx may help identify a supraglottic source.

Regardless of the bleeding source, initial steps for managing massive hemoptysis should focus on hemodynamic stabilization. The primary goal is to secure the airway, as intubation would become challenging with ongoing hemorrhage. Also, as intra-bronchial bleeding is most common from the left lung, attempts should be made to isolate the non-bleeding right lung by using a single-lumen or double-lumen endotracheal tube. When using single-lumen endotracheal tubes, one should aim for a large lumen (8.5-9 mm internal diameter), as they allow the use of a flexible bronchoscope and bronchial blockers without occluding the lumen or limiting ventilation/oxygenation to the non-bleeding lung. A double-lumen endotracheal tube can provide single lung ventilation and isolation; however, placing them is time-consuming and requires expertise, even during a controlled nonemergency situation. Most importantly, the small lumens of double-lumen endotracheal tubes can easily be obstructed with blood and clots, making any access to the airway nearly impossible. Hence, using double-lumen tubes is not recommended for treating massive hemoptysis [6-9].

Diagnostic workup for massive hemoptysis should begin with ruling out non-pulmonary causes of bleeding, such as epistaxis or hematemesis. Primary laboratory studies, including coagulation parameters, should be evaluated such that appropriate reversal may be employed. This is particularly important when managing massive hemoptysis. Fiberoptic bronchoscopy is the initial diagnostic procedure of choice because it can be performed at the bedside, is readily available, and is often successful at localizing the bleeding site if performed while the patient is still bleeding [5]. Clot extraction and recovery of airway patency can be accomplished using therapeutic bronchoscopes with larger working channels. The availability of interventional pulmonology and/or otolaryngology teams on-site allowed timely diagnosis and management of our patients.

The imaging modalities pertinent to evaluating massive hemoptysis include chest radiography, CT angiography (Figure 3), and thoracic aortography with provision for bronchial artery embolization. Some authors suggest that CT angiography can replace bronchoscopy as a first-line investigational approach because of its higher diagnostic yield, while others advocate it as complementary to fiberoptic bronchoscopy for bleeding site identification. Among patients who do not require fiberoptic bronchoscopy for airway management, chest radiography, and CT angiography are highly informative to guide the approach to bronchial artery embolization. In the setting of true massive and life-threatening hemoptysis, however, fiberoptic bronchoscopy should be the first-line approach [10].

Localized bleeding within the lingula shortly after completion of an LAAO device insertion is more likely venous in origin. For our patients, CT angiography identified the bleeding coming from the left lung but was unable to localize the source, while bronchoscopy was able to localize the source of bleeding and help direct the treatment approach.

## Conclusions

To conclude, periprocedural hemoptysis after LAAO device placement can often be attributed to coagulopathy and pulmonary venous bleed secondary to trauma induced by device insertion. Although venous bleeds are rare, they may be encountered more frequently given the rise in the number of LAAO device procedures and the ever-evolving appearance of new devices and techniques. Anticipation along with a swift and time-sensitive plan of action (e.g., bronchoscopy, CT angiogram, one-lung ventilation, activation of interventional pulmonology, and thoracic surgery teams) is warranted for managing this rare but life-threatening complication to achieve the best possible outcome. To our knowledge, ours is the first case report describing two patients who experienced hemoptysis from pulmonary venous hemorrhage as a complication of LAAO device insertion, as well as the successful management of this serious complication.
